# Knowledge and Attitudes of Amateur Male Football Players Toward Concussion in the United Kingdom

**DOI:** 10.7759/cureus.91002

**Published:** 2025-08-25

**Authors:** Robert Adams

**Affiliations:** 1 Sports and Exercise Medicine, Faculty of Biological Sciences, University of Leeds, Leeds, GBR

**Keywords:** amateur football, concussion, concussion education, rockas, symptom reporting

## Abstract

Introduction and aim

Sports-related concussion is a recognized public health concern due to its acute and long-term neurological consequences. While most research has focused on elite athletes, amateur male footballers, who comprise the majority of UK football participants, remain under-investigated. This study aimed to evaluate concussion knowledge and attitudes among adult amateur male football players in the United Kingdom using the validated Rosenbaum Concussion Knowledge and Attitudes Survey (RoCKAS), and to explore the relationship between knowledge and attitudes.

Methods

A cross-sectional survey was distributed online between February and June 2025. Eligible participants were male amateur football players aged 18 or above. The survey assessed knowledge via the Concussion Knowledge Index (CKI) and attitudes via the Concussion Attitudes Index (CAI). Descriptive statistics and Pearson correlation were used to analyze responses.

Results

Fifty-seven respondents completed the survey. The mean CKI score was 19.92 (SD 3.58), and the mean CAI score was 63.47 (SD 10.43), indicating generally good knowledge and positive attitudes. However, key misconceptions persisted, and 38.6% (n=22) reported that they would continue playing despite symptoms. No significant correlation was found between knowledge and attitudes (r=0.03, p=0.83).

Conclusion

While factual understanding was generally strong, key misconceptions and risk-tolerant behaviors persist. The lack of association between knowledge and attitudes reinforces existing concerns that education alone may be insufficient to change behavior. Multifactorial, context-sensitive strategies that address psychosocial and cultural influences may be more effective in improving concussion safety within amateur football.

## Introduction

Sports-related concussion (SRC) is defined as a traumatic brain injury caused by biomechanical forces, characterized by a complex pathophysiological process affecting the brain [1]. Symptoms typically involve physical, cognitive, and emotional domains, including headache, dizziness, memory disturbance, irritability, and mood changes [2]. Although most concussions resolve without persistent sequelae [3], accumulating evidence indicates that multiple concussive events may increase the risk of cognitive impairment, psychiatric disorders, and neurodegenerative conditions such as chronic traumatic encephalopathy (CTE) [4]. As awareness of these long-term risks grows, greater emphasis has been placed on early recognition, appropriate management, and prevention strategies to protect athlete welfare. Sports-related concussion is now a major topic of media coverage and public debate, particularly following high-profile cases of neurodegenerative conditions in retired professional athletes across sports, including American football, rugby union, and boxing [5].

Football, as the most widely played amateur sport in the United Kingdom, is highly relevant within the context of sports-related concussion, particularly given its large participant base and frequent exposure to head contact [6]. Heading the ball, aerial challenges, player collisions, and accidental head-to-head contact all contribute to cumulative exposure to concussive and sub-concussive forces. Emerging evidence suggests that repetitive sub-concussive impacts, such as frequent heading, may contribute to cumulative neurological effects even in the absence of overt clinical symptoms [7,8]. In response to these concerns, the Football Association (FA) introduced heading restrictions for youth players from the start of the 2024/25 season, reflecting growing caution around the potential long-term consequences of repetitive head impacts [9]. While professional footballers often benefit from medical supervision, structured concussion protocols, and access to multidisciplinary care, amateur players typically compete without such safeguards, raising concerns about their ability to recognize, report, and appropriately manage concussive injuries.

Several studies have explored concussion knowledge and attitudes in elite and collegiate sporting populations, particularly in North America, where structured concussion education is more established [10]. Despite these efforts, persistent misconceptions continue to be reported. High rates of symptom under-recognition and underreporting have been observed in the literature. Among North American collegiate athletes, 70% of soccer (n=56) and American football players who experienced symptoms consistent with concussion failed to recognize these as concussive episodes, with misunderstanding and misconceptions of symptoms also remaining common [11,12].

In the United Kingdom, most existing research has focused on elite male and elite female football cohorts, with limited attention paid to the amateur male game. Variability in knowledge and attitudes has been demonstrated among professionals in English second-division clubs [13]. Formal concussion education remains inconsistent, with only 48% of elite (n=57) English footballers having received structured education on concussion management, and even lower rates reported among female players [14]. Despite the existence of structured protocols in professional leagues, compliance with recommended concussion management practices remains suboptimal, even in English Championship teams with access to medical resources [15]. These findings suggest that gaps in concussion knowledge and practice may not be limited to resource-constrained amateur settings. More recently, knowledge and attitudes in elite women’s football in England have been explored, again highlighting ongoing knowledge deficits and reinforcing the need for targeted educational interventions [16].

Beyond knowledge deficits, psychosocial factors play a significant role in the underreporting of concussion symptoms. Athletes may consciously choose not to disclose symptoms due to fear of losing playing time, disappointing teammates, or appearing weak [17,18]. This culture of playing through injury has been widely reported across multiple sports and may be particularly pronounced in amateur contexts where formal medical oversight is minimal. Pressures related to team selection, peer influence, and self-management have also been identified as important barriers to disclosure [19].

Despite growing literature in elite settings, adult amateur male football remains comparatively under-investigated, particularly within the United Kingdom. This is of concern given that amateur participants represent the largest proportion of football players, yet typically compete without access to formal medical support, structured education programs, or regulated return-to-play protocols. These factors may contribute to continued knowledge deficits, potentially compromising both acute management and longer-term welfare following head injury. Improving concussion knowledge and attitudes at the amateur level is therefore critical to supporting early recognition, timely reporting, and safe management of head injuries. Enhancing understanding within this population may inform targeted education strategies and contribute to evidence-based policy development to safeguard player welfare.

This study aimed to evaluate concussion knowledge and attitudes among adult amateur male football players in the United Kingdom using the validated Rosenbaum Concussion Knowledge and Attitudes Survey (RoCKAS). This study assessed both general knowledge and symptom recognition through the Concussion Knowledge Index (CKI) and attitudes toward concussion reporting and management through the Concussion Attitudes Index (CAI). It was hypothesized that overall concussion knowledge would be limited within this population, and that greater knowledge would be associated with more favorable attitudes toward concussion management and reporting behaviors.

## Materials and methods

Study design

This was a cross-sectional, observational study designed to evaluate concussion knowledge and attitudes among adult amateur male football players in the United Kingdom. This study used an anonymous online questionnaire administered over a four-month period.

Participants

Eligible participants were male individuals aged 18 years or older who were actively playing in UK amateur football leagues (defined as not being paid to play in the league) at the time of recruitment. Exclusion criteria included prior professional football experience or current/prior healthcare-related qualifications (including medicine, nursing, physiotherapy, paramedicine, sports science, or psychology), to minimize bias associated with specialist knowledge of concussion.

Recruitment was conducted between 26 February and 20 June 2025. A dual recruitment strategy was employed. First, league secretaries across amateur football leagues in the United Kingdom were contacted via email and invited to distribute the survey to eligible players. Second, a snowball sampling method was used, encouraging players to circulate the survey within their networks. Eligibility was confirmed through screening questions at the start of the survey.

Survey instrument

Concussion knowledge and attitudes were assessed using a previously modified version of the Rosenbaum Concussion Knowledge and Attitudes Survey (RoCKAS), a validated tool widely applied in sport-related concussion research [20]. This version included an adapted symptom recognition scale, which has demonstrated improved internal consistency (Cronbach’s alpha 0.83 versus 0.71) [21] and has been validated in football-playing populations [13,16,22].

The Concussion Knowledge Index (CKI) consisted of 25 true/false items assessing general knowledge and symptom recognition. Each correct response was awarded one point (range: 0-25). The Concussion Attitudes Index (CAI) included 15 items rated on a five-point Likert scale, with higher scores reflecting safer attitudes toward concussion reporting and management (range: 15-75).

Additional questions were included to gather demographic and experiential data, such as years of football participation, previous concussion diagnosis, and whether the participant had received any form of formal concussion education. If education had been received, participants were asked to indicate the setting (e.g., club, school, or governing body). To ensure response validity, three internal validity items were embedded in the questionnaire. Participants answering one or more of these items incorrectly were excluded from the analysis.

Data collection

The survey was hosted on the Jisc Online Surveys platform. Data collection commenced on February 26, 2025, and concluded on June 20, 2025. League secretaries who agreed to assist were sent the survey link and asked to distribute it via team email lists or communication apps. A follow-up reminder was sent eight weeks into the recruitment period to encourage further responses.

The survey was voluntary, anonymous, and took approximately 10-15 minutes to complete. No personal identifiers were collected. Participants could withdraw at any point prior to final submission. Only completed responses that passed the internal validity screen were included in the final analysis.

Data analysis

Survey data were exported into IBM SPSS Statistics version 30 (Armonk, NY: IBM Corp.) for analysis. Descriptive statistics (means, standard deviations, and frequencies) were calculated for all variables. The CKI and CAI were treated as continuous variables. The Kolmogorov-Smirnov test was used to assess normality, appropriate for a sample size exceeding 50. As both variables approximated a normal distribution, Pearson correlation analysis was used to assess the relationship between CKI and CAI scores.

## Results

Participant characteristics

A total of 57 participants completed the survey. Of these, 26% (n=15) reported a previous diagnosis of concussion. The majority (87%; n=50) had been playing amateur football for over five years, and 17.5% (n=10) participants indicated that they had received some form of formal concussion education. No participants were excluded on the basis of failing the internal validity questions. Participant characteristics are summarized in Table 1.

**Table 1:** Participant characteristics.

Characteristics	n (%)
Total participants	57
Previous concussion (%)	15 (26.3%)
>5 years playing football	50 (87.7%)
Formal concussion education	10 (17.5%)

Concussion Knowledge Index

The mean Concussion Knowledge Index (CKI) score was 19.92 (SD 3.58) out of 25. All respondents (100%; n=57) correctly reported that loss of consciousness is not required for the diagnosis of concussion, and 94.7% (n=54) identified headache as a common symptom. Additionally, 80.7% (n=46) reported that sustaining one concussion increases the likelihood of subsequent concussions, and 77.2% (n=44) responded that a concussion can occur without direct head contact.

The item most frequently answered incorrectly was “weakness in neck range of motion,” with 64.9% (n=37) of participants endorsing it as a concussion symptom. A total of 59.6% (n=34) correctly stated that symptoms usually resolve within 10 days, and 45.6% (n=26) correctly responded that brain imaging typically does not show visible abnormalities following concussion. A full breakdown of itemized responses to CKI sections one, two, and five is available in Tables 2, 3.

**Table 2:** CKI sections one and two. CKI: Concussion Knowledge Index

Variables	All responses		Previous concussion		No previous concussion		Previous education		No previous education	
	N correct	% correct	N correct	% correct	N correct	% correct	N correct	% correct	N correct	% correct
Section 1										
There is a possible risk of death if a second concussion occurs before the first one has healed.	48	84.2	13	86.7	35	83.3	8	80	40	85.1
People who have had one concussion are more likely to have another concussion.	46	80.7	12	80	34	81.0	8	80	38	80.9
In order to be diagnosed with a concussion, you have to be knocked out.	57	100	15	100	42	100.0	10	100	47	100
A concussion can only occur if there is a direct hit to the head.	44	77.2	12	80	32	76.2	8	80	36	76.6
Being knocked unconscious always causes permanent damage to the brain.	43	75.4	13	86.7	31	73.8	8	80	35	74.5
Symptoms of a concussion can last for several weeks.	57	100	15	100	42	100.0	10	100	47	100
Sometimes, a second concussion can help a person remember things that were forgotten after the first concussion.	47	82.5	15	100	32	76.2	8	80	39	83
After a concussion occurs, brain imaging (e.g., CT scan, MRI, X-Ray, etc.) typically shows visible physical damage (e.g., bruise, blood clot) to the brain.	31	54.4	11	73.3	20	47.6	4	40	27	57.4
If you sustain one concussion and you have never had a concussion before, you will become less intelligent.	55	96.5	15	100	40	95.2	9	90	46	97.9
After 10 days, symptoms of a concussion are usually completely gone.	34	59.6	7	46.7	27	64.3	6	60	28	59.6
After a concussion, people can forget who they are and not recognize others, yet remain perfect in every other way.	46	80.7	12	80	34	81.0	8	80	38	80.9
Concussions can sometimes lead to emotional disruption.	55	96.5	14	93.3	41	97.6	10	100	45	95.7
An athlete who gets knocked out after getting a concussion experiences a coma.	46	80.7	13	86.7	33	78.6	8	80	38	80.9
There is rarely a risk to long-term health and well-being from multiple concussions.	49	86	13	86.7	36	85.7	9	90	40	85.1
Section 2										
It is likely that player Q’s concussion will affect his long-term health and well-being.	45	78.9	15	100.0	30	71.4	8	80	37	78.7
It is likely that player X’s concussion will affect his long-term health and well-being.	48	84	13	86.7	35	83.3	9	90	39	83
Even though player F is still experiencing the effects of the concussion, her performance will be the same as it would be had she not suffered a concussion.	48	84	13	86.7	35	83.3	9	90	39	83

**Table 3:** CKI section five, the 16-item symptom recognition checklist. CKI: Concussion Knowledge Index

Variables	All responses		Previous concussion		No previous concussion		Previous education		No previous education	
Symptoms	N correct	% selected	N correct	% selected	N correct	% selected	N correct	% selected	N correct	% selected
Abnormal sense of smell	44	22.8	12	20	32	43.9	9	10	35	46.8
Abnormal sense of taste	41	28.1	11	26.7	30	47.4	8	20	33	51.1
Amnesia	43	75.4	11	73.3	32	56.1	7	70	36	76.6
Blurred vision	52	91.2	13	86.7	39	68.4	9	90	43	91.5
Black eye	44	22.8	13	13.3	31	45.6	10	0	34	27.7
Chest pain	46	19.2	13	13.3	33	42.1	10	0	36	23.4
Confusion	49	86	12	80	37	64.9	10	100	39	83
Dizziness	54	94.7	15	100	39	68.4	10	100	44	93.6
Headache	54	94.7	15	100	39	68.4	10	100	44	93.6
Loss of consciousness	50	87.7	12	100	38	66.7	10	100	40	85.1
Nausea	47	82.5	12	80	35	61.4	8	80	39	83
Nosebleed	38	33.3	11	26.7	27	52.6	7	30	31	34
Numbness/tingling in the upper extremity	27	52.6	9	40	18	68.4	8	20	19	59.6
Sharp burning pain in the neck	46	19.3	14	6.7	32	43.9	10	0	36	23.4
Sleep disturbances	39	68.4	9	60	30	52.6	5	50	34	72.3
Weakness in neck range of motion	20	64.9	5	66.7	15	73.7	5	50	15	68

Concussion Attitudes Index

The mean Concussion Attitudes Index (CAI) score was 63.47 (SD 10.43) out of 75. The item with the highest rate of safe responses was “I feel that concussions are less important than other injuries” (94.7%; n=54) and “I feel that manager A made the right decision to keep player R out of the game” (94.7%; n=54). The item “I would continue playing a sport while also having a headache that resulted from a minor concussion” was endorsed unsafely by 38.6% (n=22) of participants. No other individual CAI items showed a high proportion of unsafe responses. A full breakdown of CAI responses can be seen in Table 4. A full breakdown of item-level responses is presented in the table in appendix.

**Table 4:** Concussion Attitudes Index responses.

Variables	All responses		Previous concussion		No previous concussion		Previous education		No previous education	
	N safe answers	% safe answers	N safe answers	% safe answers	N safe answers	% safe answers	N safe answers	% safe answers	N safe answers	% safe answers
Section 3										
I would continue playing a sport while also having a headache that resulted from a minor concussion.	35	61.4	10	66.7	25	59.5	7	70	28	59.6
I feel that coaches need to be extremely cautious when determining whether a player should return to play.	52	91.2	13	86.7	39	92.9	9	90	43	91.5
I feel that concussions are less important than other injuries.	54	94.7	15	100	39	92.9	8	80	46	97.9
I feel that a player has a responsibility to return to a game, even if it means playing while still experiencing symptoms of concussion.	50	87.7	15	100	35	83.3	8	80	42	89.4
I feel that a player who is knocked unconscious should be taken to the Emergency Department (A&E).	51	89.5	15	100	36	85.7	10	100	41	87.2
Section 4										
I feel that manager A made the right decision to keep player R out of the game.	54	94.7	15	100	39	92.9	8	80	46	97.9
Most players would feel that manager A made the right decision to keep player R out of the game.	35	61.4	15	100	20	47.6	10	100	25	53.2
I feel that player M should have returned to play during the first game of the season.	53	93	15	100.0	38	90.5	8	80	45	95.7
Most players would feel that player M should have returned to play during the first game of the season.	42	73.7	15	100	27	64.3	10	100	32	68.1
I feel that player Q should have returned to play during the semi-final match.	50	87.7	12	80	38	90.5	8	80	42	89.4
Most players would feel that player Q should have returned to play during the semi-final match.	37	64.9	14	93.3	23	54.8	6	60	31	66
I feel that the physiotherapist, rather than the player, should make the decision about returning player R to play.	43	75.4	11	73.3	32	76.2	7	70	36	76.6
Most physiotherapists would feel that the physiotherapist, rather than the player, should make the decision about returning player R to play.	47	82.5	11	73.3	36	85.7	10	100	37	78.7
I feel that athlete H should tell their coach about the symptoms.	52	91.2	14	93.3	38	90.5	8	80	44	93.6
Most physiotherapists would feel that athlete H should tell their coach about the symptoms.	49	86	14	93.3	35	83.3	9	90	40	85.1

Association between CKI and CAI scores

**Figure 1:**
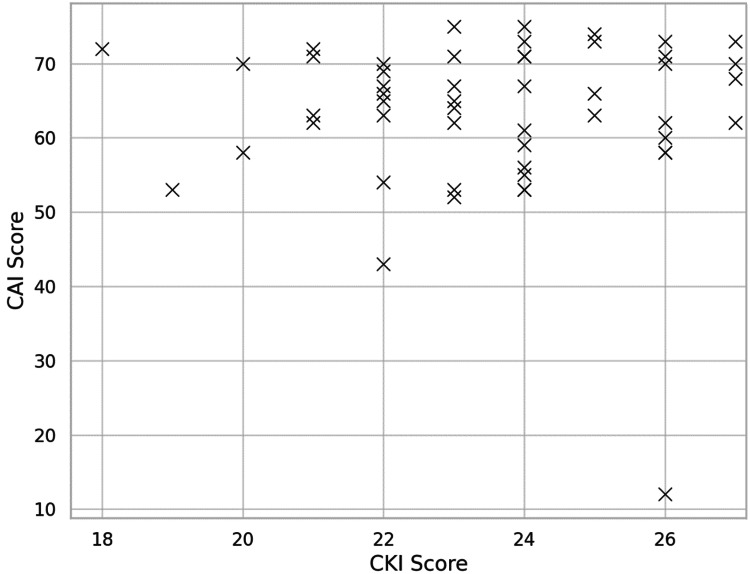
Scatterplot showing the relationship between Concussion Knowledge Index (CKI) and Concussion Attitudes Index (CAI) scores (r = -0.03, p=0.83; n=57).

**Figure 2:**
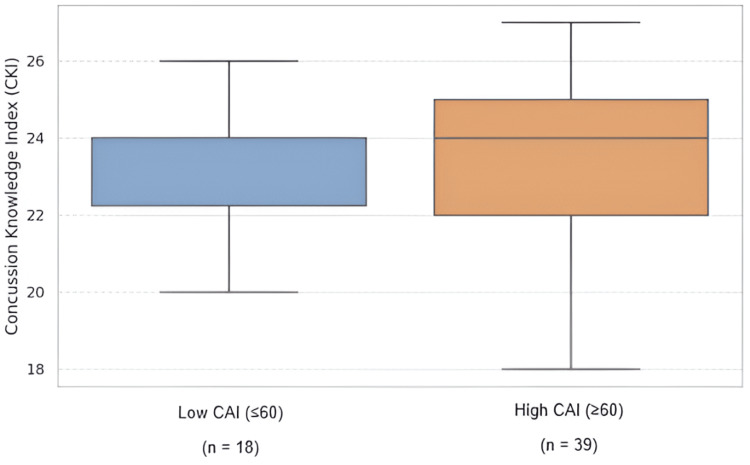
Distribution of concussion knowledge (CKI) scores in participants with low versus high attitude (CAI) scores (n=57).

Kolmogorov-Smirnov tests indicated that CAI scores were normally distributed (p=0.288), while CKI scores showed a mild deviation from normality (p=0.026). Pearson correlation analysis was used due to the approximate normality and robustness of the test to minor non-normality in moderate sample sizes. As shown in Figures 1, 2, the analysis found no significant correlation between CKI and CAI scores (r = -0.03, p=0.83).

## Discussion

Study aims and summary of key findings

The aim of this study was to evaluate concussion knowledge and attitudes among adult amateur male football players in the United Kingdom using the validated Rosenbaum Concussion Knowledge and Attitudes Survey (RoCKAS). Specifically, it assessed knowledge and symptom recognition through the Concussion Knowledge Index (CKI), attitudes toward concussion reporting and management through the Concussion Attitudes Index (CAI), and the association between knowledge and attitudes. It was hypothesized that concussion knowledge would be limited within this population and that greater knowledge would be associated with safer attitudes.

A total of 57 participants met the inclusion criteria and completed the survey. The mean age of respondents was 34.68 years (SD: 11.7). The mean CKI score was 19.92 (SD: 3.58) out of 25, suggesting generally good factual knowledge. All participants (100%; n=57) correctly identified that loss of consciousness is not required for concussion diagnosis, and 94.7% (n=54) recognized headache as a common symptom. Additionally, 80.7% (n=46) acknowledged that prior concussion increases susceptibility to future concussions. However, misconceptions persisted. Most notably, 64.9% (n=37) incorrectly identified "weakness in neck range of motion" as a symptom of concussion.

Attitudes toward concussion were generally positive, with a mean CAI score of 63.47 (SD: 10.43) out of 75. Most respondents (94.7%; n=54) disagreed with the statement that concussions are less important than other injuries, and 91.2% (n=52) indicated that athletes experiencing symptoms should inform their coach. However, 38.6% (n=22) of participants stated they would continue playing while experiencing a concussion-related headache. No significant association was observed between knowledge and attitudes (r = -0.03, p=0.83), indicating that higher knowledge did not necessarily translate into safer behavioral intentions.

Concussion knowledge in context

The level of concussion knowledge observed in this study aligns with prior RoCKAS-based research in elite and sub-elite football populations. Moderate knowledge, alongside persistent misconceptions, has been reported among English Championship footballers [13], as well as among English professional female players with access to formal concussion education programs [16]. The present findings extend these trends to the amateur male population, which typically competes without regular medical oversight.

The misconception regarding neck symptoms is consistent with prior studies using modified RoCKAS symptom scales that include distractor items such as “neck weakness” [21]. The continued endorsement of such non-specific symptoms may reflect diagnostic confusion between concussion and concurrent musculoskeletal or cervical spine injuries, which can co-occur but are clinically distinct.

Broader difficulties with symptom recognition have been well documented. Among American collegiate athletes, up to 70% failed to correctly identify a previous concussion [11,12]. The present results suggest that these challenges extend across both elite and amateur sport, underscoring the need for improved education around concussion symptom patterns and variability.

Attitudes toward concussion

Attitudes toward concussion disclosure and management were largely favorable, with high agreement around symptom reporting and recognition of concussion severity. This is encouraging, as attitudes are well-established predictors of reporting behavior. However, previous studies have shown that even when knowledge is good, athletes may fail to report symptoms due to cultural, interpersonal, or psychological barriers. Among US collegiate athletes, underreporting has been linked to fears of losing playing time or letting down teammates [17,18]. Similar psychosocial pressures have been identified in amateur and elite rugby settings [19,23].

The finding that 38.6% (n=22) of respondents would continue playing despite symptoms suggests a persistent risk-tolerant subset. This aligns with research showing that knowledge alone may not be sufficient to change behavior, especially in environments with strong sporting norms [11,12]. The absence of a significant knowledge-attitudes correlation in the present study reinforces this view and underlines the importance of addressing sociocultural influences alongside factual education. Table 5 compares the mean CKI and CAI scores in this study with those reported in other RoCKAS-based research.

**Table 5:** Comparison of current study to other studies that used the RoCKAS survey. RoCKAS: Rosenbaum Concussion Knowledge and Attitudes Survey; CKI: Concussion Knowledge Index; CAI: Concussion Attitudes Index

Studies	Sports	N		Mean CKI score	Mean CAI score	Correlation coefficient
Travis et al. (2024) [23]	American football		236	21 (SD 2.1)	55.6 (SD 6.1)	-
							Kraak et al. (2018) [22]	University rugby players		180	18.8 (SD 2.4)	60.98 (SD 6.32)	0.14
Gallagher and Falvey (2017) [24]	Gaelic footballers		70	18.7 (SD 2.2)	60.3 (SD 6.9)	-
							Shafik et al. (2024) [16]	English professional female footballers		111	20.53 (SD 2.31)	63.28 (6.33)	0.2
Williams et al. (2016) [13]	English men’s professional players		26	16.4 (SD 2.9)	59.6 (SD 8.5)	-							
							Current study	UK amateur male footballers		57	19.92 (3.58)	63.47 (SD 10.43)	-0.03

Knowledge-attitudes relationship

The hypothesis that greater knowledge would be associated with more favorable attitudes was not supported. This aligns with previous research suggesting that knowledge and attitudes, while conceptually linked, often show weak or inconsistent correlations [25]. Several factors may underlie this disconnect. Even when athletes are aware of concussion risks, emotional, motivational, or situational pressures may override this knowledge at the moment of decision-making.

Behavioral science models support this view. Frameworks such as the Health Belief Model and Theory of Planned Behavior highlight the importance of perceived severity, social norms, and self-efficacy in driving behavior [26,27]. In amateur football, where medical supervision is minimal and decisions are often made by athletes or coaches themselves, these social and contextual factors likely exert considerable influence. Recent studies have shown that information-based education has a limited impact unless paired with strategies to shift norms, model behavior, and strengthen support networks [28,29].

Educational initiatives that go beyond knowledge transfer - including peer-led modelling, coach reinforcement, and targeted disruption of risk-tolerant norms - may prove more effective, particularly in grassroots football environments with minimal institutional safeguards.

Limitations

Several limitations should be acknowledged when interpreting the findings of this study. Firstly, the reliance on self-reported data via an online questionnaire introduces the potential for response bias, including social desirability bias and recall inaccuracies. Although the survey was anonymized to mitigate these effects, it remains possible that participants provided answers they perceived as socially acceptable rather than reflective of their true beliefs or behaviors. Secondly, while the RoCKAS instrument is validated and widely used, its structured format may limit the exploration of nuanced or context-dependent concussion attitudes, particularly in the diverse and informal settings of amateur football. The cross-sectional design of the study also precludes any inference of causality between knowledge and attitudes, and does not account for how these constructs may evolve over time or in response to specific experiences such as prior concussions or education. Furthermore, the sample comprised exclusively adult male amateur footballers in the United Kingdom, which restricts the generalizability of findings to other groups, including female athletes, youth populations, and those participating in other levels or types of sport. Finally, the recruitment strategy, which relied in part on voluntary participation and snowball sampling, may have led to selection bias. Individuals with a pre-existing interest in concussion or sports medicine may have been more likely to participate, potentially inflating knowledge and attitude scores and limiting representativeness.

## Conclusions

This study provides important insight into concussion knowledge and attitudes among adult male amateur footballers in the United Kingdom, a population underrepresented in existing literature. Participants demonstrated broadly accurate knowledge, yet misconceptions in symptom recognition and the endorsement of risk-tolerant behaviors persist. The lack of a significant correlation between knowledge and attitudes suggests that factual understanding alone may be insufficient to influence behavioral intent.

These findings highlight the limitations of education-only interventions and support the need for multifaceted, context-sensitive strategies informed by behavioral science. Future efforts to improve concussion safety in all playing groups of football should account for the sociocultural environment in which players make decisions, and prioritize interventions that enhance not only knowledge but also self-efficacy, normative beliefs, and situational readiness to report.

## Appendices

**Table 6:** The complete version of the survey received by participants.

Assessing concussion knowledge and attitudes in amateur men’s football in the United Kingdom: a cross-sectional study
Thank you for taking the time to participate in this research study, which aims to assess the knowledge and attitudes toward concussion of amateur male football players in the United Kingdom.
This survey should take around 10 minutes to complete.
Your responses will remain anonymous, and no identifiable personal information will be collected. Participation is entirely voluntary, and you can exit the survey at any time.
Please read the participant information document before proceeding, which can be viewed here:
Link to participant information
If you agree to participate and meet all criteria as mentioned in the information sheet, please proceed by selecting "I consent to participate in this study" below
I consent to participate in this study
What is your age?
How long have you been playing amateur football?
Less than a year | 1-3 years | 3-5 years | 5+ years
Have you ever been diagnosed with a concussion before?
Yes | No | Unsure
Have you ever received formal education about a concussion?
Yes | No
If you answered yes to the above question, where did you receive your formal education about concussion?
My club provided various training options, including online courses such as the FA Concussion Module and FIFA Concussion Awareness Course, as well as higher education opportunities like university coaching/refereeing qualifications, workplace training, or others.
What is the name of the team you played for most recently? This will not be included in the data analysis (you may choose to leave blank).
There is a possible risk of death if a second concussion occurs before the first one has healed.
True | False
People who have had one concussion are more likely to have another concussion.
True | False
In order to be diagnosed with a concussion, you have to be knocked out.
True | False
A concussion can only occur if there is a direct hit to the head.
True | False
Being knocked unconscious always causes permanent damage to the brain.
True | False
Symptoms of a concussion can last for several weeks.
True | False
Sometimes, a second concussion can help a person remember things that were forgotten after the first concussion.
True | False
After a concussion occurs, brain imaging (e.g., CT scan, MRI, X-ray, etc.) typically shows visible physical damage (e.g., bruise, blood clot) to the brain.
True | False
If you receive one concussion and you have never had a concussion before, you will become less intelligent.
True | False
A square is a 4-sided shape.
True | False
After 10 days, symptoms of a concussion are usually completely gone.
True | False
After a concussion, people can forget who they are and not recognize others but be perfect in every other way.
True | False
Concussions can sometimes lead to emotional disruption.
True | False
An athlete who gets knocked out after getting a concussion is experiencing a coma.
True | False
There is rarely a risk to long-term health and well-being from multiple concussions.
True | False
While playing in a game, player A and player B collide with each other and both suffer a concussion. Player A has never had a concussion before, and player B has had 4 concussions in the past.
It is likely that player A's concussion will affect his long-term health and well-being.
True | False
It is likely that player B's concussion will affect his long-term health and well-being.
True | False
A triangle has 6 sides.
True | False
Player C suffered a concussion in a game. She continued to play in the same game despite the fact that she continued to feel the effects of the concussion.
Even though player C is still experiencing the effects of concussion, her performance will be the same as it would be had she not suffered a concussion.
True | False
2 + 2 = 5
True | False
Which of the following are symptoms of a concussion? You may select as many options as you like.
Abnormal sense of smell | Abnormal sense of taste | Amnesia | Blurred vision | Black eye | Chest pain | Confusion | Dizziness | Headache | Loss of consciousness | Nausea | Nosebleed | Numbness/tingling in the upper extremity | Sharp burning pain in the back | Sleep disturbances | Weakness of neck range of motion
I would continue playing a sport while also having a headache that resulted from a minor concussion.
Strongly disagree | Disagree | Unsure | Agree | Strongly agree
I feel that coaches need to be extremely cautious when determining whether a player should return to play.
Strongly disagree | Disagree | Unsure | Agree | Strongly agree
I feel that concussions are less important than other injuries.
Strongly disagree | Disagree | Unsure | Agree | Strongly agree
I feel that a player has a responsibility to return to a game, even if it means playing while still experiencing symptoms of concussion.
Strongly disagree | Disagree | Unsure | Agree | Strongly agree
I feel that a player who is knocked unconscious should be taken to the Emergency Department (A&E)
Strongly disagree | Disagree | Unsure | Agree | Strongly agree
Scenario 1:
Player R suffers a concussion during a game. Manager A decides to keep this player (R) out of the game. Player R's team lost the game.
I feel that manager A made the right decision to keep player R out of the game.
Strongly disagree | Disagree | Unsure | Agree | Strongly agree
Most players would feel that manager A made the right decision to keep player R out of the game.
Strongly disagree | Disagree | Unsure | Agree | Strongly agree
Scenario 2:
Player M suffers a concussion during the first game of the season. Player Q suffered a concussion of the same severity during a semi-final match. Both players had persisting symptoms.
I feel that player M should have returned to play during the first game of the season.
Strongly disagree | Disagree | Unsure | Agree | Strongly agree
Most players would feel that player M should have returned to play during the first game of the season.
Strongly disagree | Disagree | Unsure | Agree | Strongly agree
I feel that player Q should have returned to play during the semi-final match.
Strongly disagree | Disagree | Unsure | Agree | Strongly agree
Most players would feel that player Q should have returned to play during the semi-final match.
Strongly disagree | Disagree | Unsure | Agree | Strongly agree
Scenario 3:
Player R suffers a concussion. Player R's team has a physiotherapist.
I feel that the physiotherapist, rather than the player, should make the decision about returning player R to play.
Strongly disagree | Disagree | Unsure | Agree | Strongly agree
Most physiotherapists would feel that the physiotherapist, rather than the player, should make the decision about returning player R to play.
Strongly disagree | Disagree | Unsure | Agree | Strongly agree
Scenario 4:
Athlete H suffered a concussion and has a game in 2 hours. They are still experiencing symptoms of concussion. They know that if they mention the symptoms, their coach will keep them out of the game.
I feel that athlete H should tell their coach about the symptoms.
Strongly disagree | Disagree | Unsure | Agree | Strongly agree
Most physiotherapists would feel that athlete H should tell their coach about the symptoms.
Strongly disagree | Disagree | Unsure | Agree | Strongly agree

## References

[1] McCrory P, Feddermann-Demont N, Dvořák J (2017). What is the definition of sports-related concussion: a systematic review. Br J Sports Med.

[2] Junn C, Bell KR, Shenouda C, Hoffman JM (2015). Symptoms of concussion and comorbid disorders. Curr Pain Headache Rep.

[3] Patel H, Polam S, Joseph R (2024). Concussions: a review of physiological changes and long-term sequelae. Cureus.

[4] Manley G, Gardner AJ, Schneider KJ (2017). A systematic review of potential long-term effects of sport-related concussion. Br J Sports Med.

[5] Hume PA, Theadom A, Lewis GN, Quarrie KL, Brown SR, Hill R, Marshall SW (2017). A comparison of cognitive function in former rugby union players compared with former non-contact-sport players and the impact of concussion history. Sports Med.

[6] FA FA, The The (2024). The FA for all. https://www.thefa.com/about-football-association/for-all#:~:text=It's%20for%20everyone.,or%20disability%2C%20faith%20or%20age..

[7] Broglio SP, Eckner JT, Martini D, Sosnoff JJ, Kutcher JS, Randolph C (2011). Cumulative head impact burden in high school football. J Neurotrauma.

[8] Johnson B, Neuberger T, Gay M, Hallett M, Slobounov S (2014). Effects of subconcussive head trauma on the default mode network of the brain. J Neurotrauma.

[9] FA FA, T. 2025. Football for all [Online (2025). Heading in football. https://www.englandfootball.com/participate/learn/brain-health/heading-in-football.

[10] Kroshus E, Garnett B, Hawrilenko M, Baugh CM, Calzo JP (2015). Concussion under-reporting and pressure from coaches, teammates, fans, and parents. Soc Sci Med.

[11] Delaney JS, Lacroix VJ, Gagne C, Antoniou J (2001). Concussions among university football and soccer players: a pilot study. Clin J Sport Med.

[12] Chapman EB, Nasypany A, May J, Henry T, Hummel C, Jun HP (2018). Investigation of the Rosenbaum Concussion Knowledge and Attitudes Survey in collegiate athletes. Clin J Sport Med.

[13] Williams JM, Langdon JL, McMillan JL, Buckley TA (2016). English professional football players concussion knowledge and attitude. J Sport Health Sci.

[14] Rosenbloom C, Broman D, Chu W, Chatterjee R, Okholm Kryger K (2022). Sport-related concussion practices of medical team staff in elite football in the United Kingdom, a pilot study. Sci Med Footb.

[15] Price J, Malliaras P, Hudson Z (2012). Current practices in determining return to play following head injury in professional football in the UK. Br J Sports Med.

[16] Shafik A, Bennett P, Rosenbloom C, Okholm Kryger K, Carmody S, Power J (2024). Sport-related concussion attitudes and knowledge in elite English female footballers. Sci Med Footb.

[17] Chrisman SP, Quitiquit C, Rivara FP (2013). Qualitative study of barriers to concussive symptom reporting in high school athletics. J Adolesc Health.

[18] Kerr ZY, Register-Mihalik JK, Kroshus E, Baugh CM, Marshall SW (2016). Motivations associated with nondisclosure of self-reported concussions in former collegiate athletes. Am J Sports Med.

[19] Ryan L, Daly E, Hunzinger K (2024). Factors affecting sport-related concussion non-disclosure in women's rugby - a multi-country qualitative analysis. J Funct Morphol Kinesiol.

[20] Rosenbaum AM, Arnett PA (2010). The development of a survey to examine knowledge about and attitudes toward concussion in high-school students. J Clin Exp Neuropsychol.

[21] Valovich McLeod TC, Schwartz C, Bay RC (2007). Sport-related concussion misunderstandings among youth coaches. Clin J Sport Med.

[22] Kraak Kraak, W W, Bernardo B, Vuuren B, Loubser A, Vuuren J, Coetzee M (2018). Knowledge and attitudes amongst Stellenbosch University hostel rugby players. S Afr J Sports Med.

[23] Travis E, Scott-Bell A, Thornton C (2024). The current state of concussion knowledge and attitudes in British American Football. Phys Sportsmed.

[24] Gallagher C, Falvey E (2017). Assessing knowledge and attitudes towards concussion in Irish footballers. Br J Sports Med.

[25] Silver D, Faull-Brown R, McClusky R, Brown N, Patterson S, Stokes K, Kemp SP (2025). Concussion knowledge and attitude of English youth rugby players: the RUCKAS-YOUTH survey. BMJ Open Sport Exerc Med.

[26] Ajzen I (1991). The theory of planned behavior. Organ Behav Hum Decis Process.

[27] Rosenstock IM (1974). The health belief model and preventive health behavior. Health Educ Monogr.

[28] Foulds SJ, Hoffmann SM, Hinck K, Carson F (2019). The coach-athlete relationship in strength and conditioning: high performance athletes' perceptions. Sports (Basel).

[29] Hernandez M, Gibb JK (2020). Culture, behavior and health. Evol Med Public Health.

